# Sijing Pill modulates PGE2/EP4/PI3K-AKT pathway via gut-bone axis to treat postmenopausal osteoporosis

**DOI:** 10.3389/fmicb.2026.1786197

**Published:** 2026-03-18

**Authors:** Xiaojuan Zhu, Yufen Li, Xianting Meng, Tao Jiang, Chenhao Li, Xiujing Zhu, Zixin Yin, Junpeng Guo, Xin Su

**Affiliations:** School of Basic Medical Sciences, Changchun University of Chinese Medicine, Changchun, China

**Keywords:** gut-bone axis, microbiology, multi-omics analysis, postmenopausal osteoporosis, Sijing Pill

## Abstract

**Introduction:**

Postmenopausal osteoporosis (PMOP) represents a substantial clinical burden for aging women worldwide. Existing pharmacotherapies are frequently constrained by suboptimal efficacy, poor adherence, and adverse effects, underscoring the need for superior treatment alternatives. The Sijing Pill (SJP) has shown potential in alleviating bone loss in PMOP. However, the fundamental mechanisms underlying its therapeutic effects remain incompletely elucidated, which has impeded its clinical translation and rational application.

**Methods:**

The therapeutic effect of SJP on PMOP was first evaluated in ovariectomized (OVX) rat models using hematoxylin and eosin (H&E) staining, micro-computed tomography (μCT), and immunohistochemistry. To investigate the mechanisms, we employed an integrated strategy that combined network pharmacology, molecular docking and dynamics simulations, 16S rRNA sequencing, as well as non-targeted metabolomics coupled with MetOrigin analysis. The key predictions from these analyses were subsequently validated by Western blot and immunohistochemistry.

**Results:**

This research confirms that SJP treatment significantly alleviates abnormal weight gain and bone structural degeneration in OVX model mice. Employing an integrated multi-omics strategy, we elucidated a dual mechanism underlying the efficacy of SJP. This mechanism involves the concurrent modulation of the arachidonic acid-PGE2 metabolic axis, which ameliorates osteometabolic inflammation, alongside the remodeling of the gut microbiota, as evidenced by a decreased *Firmicutes*/*Bacteroidetes* ratio. Collectively, these factors orchestrate therapeutic effects through the gut-bone axis. Network pharmacology identified 18 bioactive components in SJP with predicted affinities for key signaling nodes, including STAT3, ESR1, and AKT1. Molecular docking confirmed high-affinity binding for pivotal pairs. Specifically, strong binding was observed between Ellipticine and COX2 (−10.6 kcal/mol) and between estrone and PTGES (−7.8 kcal/mol), implicating both in PGE2 metabolism. Functionally, SJP promoted intestinal barrier repair by upregulating ZO-1 and Occludin. In parallel, it activated the bone-specific PGE2-EP4 receptor axis and downstream PI3K-AKT signaling, thereby elucidating a direct mechanistic link through the gut-bone axis.

**Conclusion:**

Sijing Pill modulates the PGE2/EP4/PI3K-AKT signaling pathway via the gut-bone axis to aid in alleviating PMOP.

## Graphical abstract

**Figure:**
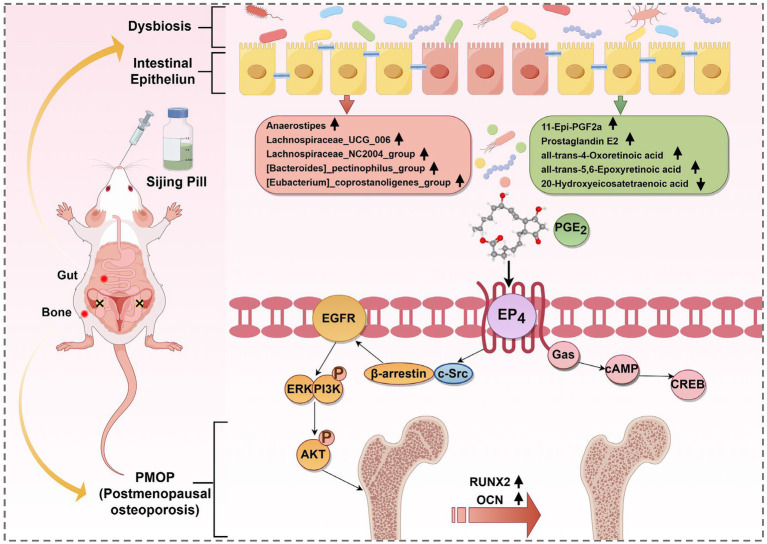

## 1 Introduction

Postmenopausal osteoporosis (PMOP), the leading subtype of primary osteoporosis in women, results from the rapid decline in estrogen levels following menopause (Xie et al., 2025; Zhao et al., 2025). Globally, PMOP affects over 50% of postmenopausal women (Li et al., 2025), accounting for approximately 200 million cases worldwide (Liu et al., 2022). The condition typically reaches its peak incidence within the first postmenopausal decade, a phase characterized by accelerated bone loss (Wu and Black, 2022). Due to its high prevalence and the fracture-driven morbidity, PMOP represents a significant global public health challenge (Chen et al., 2024). The decline in estrogen levels, a key contributor to primary osteoporosis, perturbs bone metabolism via multiple interconnected pathways (Almeida et al., 2017; Niu et al., 2024). Estrogen deficiency directly dysregulates bone metabolism, simultaneously upregulating osteoclastic bone resorption and downregulating osteoblastic bone formation (Mulinari-Santos et al., 2023; Hsu et al., 2024). The consequent net excess of resorption precipitates a high-turnover metabolic state, which uncouples bone remodeling and accelerates bone loss (Wang L. T. et al., 2023). In addition, the reduction of estrogen will also result in a persistent and systemic mild inflammation, which serves as the basis for the PMOP disease (Abildgaard et al., 2020; Mohamad et al., 2020). The inflammatory condition, characterized by elevated concentrations of pro-inflammatory cytokines such as tumor necrosis factor-alpha (TNF-*α*) and interleukin-6 (IL-6), facilitates bone resorption by concurrently stimulating osteoclast activity and inhibiting osteoblast function (Tsukasaki and Takayanagi, 2019; Wang and He, 2020; Jiang et al., 2024). An increasing number of studies indicate that the intestine assumes a crucial role in triggering this inflammation. This is due to the fact that the deficiency of estrogen impairs the intestinal lining and disrupts the microbial balance, consequently amplifying inflammatory signals and heightening oxidative stress (Kunst et al., 2023; Sahoo et al., 2023; Chu et al., 2025). The intricate pathological mechanisms outlined above underscore the ongoing challenges in achieving effective clinical management of PMOP. However, the therapeutic benefits of first-line pharmacotherapies, including bisphosphonates, SERMs, and estrogen therapy, are offset by a narrow therapeutic window and considerable adverse effects, such as gastrointestinal intolerance, thromboembolic events, and an elevated risk of breast cancer (Migliaccio et al., 2007; Cooper et al., 2012; Mirkin and Pickar, 2015; Cosman, 2018; Jackson et al., 2021). This has driven the search for safer and more comprehensive treatment strategies. In contrast, accumulating evidence highlights the therapeutic potential of Traditional Chinese Medicine (TCM) for treating PMOP by exerting multi-targeted effects on various signaling pathways to modulate bone metabolism (Du et al., 2024; Ren et al., 2024). This systems-level mechanism, unlike conventional pharmacotherapies, exerts a more comprehensive influence on bone metabolic homeostasis and contributes to a more favorable safety profile.

Sijing Pill (SJP), derived from the traditional formula Effective Formulae from Generations of Physicians, consists of four main herbal components: *Cervus nippon Temminck* (Lurong), *Cistanche deserticola* Y. C. Ma (Roucongrong), *Dioscorea polystachya Turcz*. (Shanyao), and *Poria cocos* (Schw.) Wolf (Fuling). Traditionally, this formulation is renowned for its extensive therapeutic benefits, especially in nourishing kidney yin and tonifying essence, tonifying qi of the spleen and stomach, and strengthening the sinews and bones. Recent research has started to clarify the molecular and microbiological mechanisms that underlie these effects. Mechanistically, Lurong contains insulin-like growth factor-1 (IGF-1), metabolites of testosterone, and polysaccharides that increase the abundance of *Faecalibacterium* and *Roseburia* (Wu et al., 2013; Li et al., 2014; Sui et al., 2014; You et al., 2025). The results indicate an augmentation in the formation of short-chain fatty acids (SCFAs) and a diminishment in the activity of the NF-κB/TNF-*α* pathways. This process, in turn, facilitates the proliferation of osteoblasts while attenuating the activity of osteoclasts (Liu et al., 2017; Liu Y. Y. et al., 2023). Echinacoside and acteoside, two bioactive compounds in Roucongrong, enhance *Akkermansia* and *Bifidobacterium*, elevate butyrate levels, and inhibit MAPK/NF-κB signaling, thus reducing inflammation and promoting osteogenesis (Lee et al., 2013; Wang C. et al., 2023; Yu Z. Q. et al., 2024; Huang et al., 2025). Diosgenin and mucopolysaccharides from Shanyao regulate *Lactobacillus* and *Bifidobacterium* populations, increase acetate and propionate levels, and balance RANKL/osteoprotegerin expression by inhibiting the NLRP3 inflammasome (Cheng et al., 2021; Cui et al., 2023; Tang et al., 2024; Yu W. F. et al., 2024). Moreover, the triterpenes and polysaccharides in Fuling stimulate *Bacteroides* and *Prevotella*, augment butyrate synthesis, and suppress NF-κB/STAT3 activation (Zou et al., 2021; Lai et al., 2025).

Despite the progress in characterizing the microbial regulation by individual SJP components, the systems-level mechanisms governing the synergistic actions of the full formulation have yet to be defined. In this study, our objective is to investigate the contribution of SJP to the treatment of PMOP. We primarily utilize omics and bioinformatics approaches to explore the underlying biological processes. Moreover, the study aims to identify its key active compounds, therapeutic targets, and relevant regulatory pathways. The results will offer a robust basis for the clinical application of SJP in the management of PMOP.

## 2 Materials and methods

### 2.1 Identification of active ingredients and targets in SJP

A systematic search was conducted in the TCMSP^1^, Herb^2^, and BATMAN-TCM^3^ databases to identify the active constituents and potential therapeutic targets of SJP, focusing on compounds linked to Lurong, Roucongrong, Shanyao, and Fuling. The identified compounds were subsequently assessed utilizing the SwissADME platform, conforming to the Lipinski rule of five and TCMSP standards for oral bioavailability (OB > 30%) and drug-likeness (DL ≥ 0.18) (Afridi et al., 2024; Ying et al., 2025). Potential therapeutic targets for these substances were identified utilizing the TCMSP and Swiss Target Prediction databases^4^.

### 2.2 Screening of targets related to PMOP

Genes associated with PMOP were systematically extracted utilizing the keyword “postmenopausal osteoporosis” across several databases, including GeneCards^5^, OMIM^6^, PharmGKB^7^, and TTD^8^. Following the removal of duplicate entries and the application of a relevance score threshold of ≥1 through weighted evaluation, genes pertinent to PMOP were identified. Subsequently, the overlapping target genes between the SJP component and PMOP-related genes were identified and visualized. Moreover, a protein–protein interaction (PPI) network was constructed for these shared genes by utilizing the STRING database (confidence score ≥ 0.9^9^). Core genes within this network were identified employing the CytoNCA plug-in within Cytoscape (v3.10.2).

### 2.3 Functional enrichment analysis

The functional enrichment analysis of Gene Ontology (GO) and Kyoto Encyclopedia of Genes and Genomes (KEGG) pathways was performed using the R package “clusterProfiler,” with emphasis on the shared genes linked to both drugs and diseases.

### 2.4 Medicine and reagents

SJP consists of Lurong (2 g), Roucongrong (10 g), Shanyao (30 g), and Fuling (15 g), all obtained from the Affiliated Hospital of Changchun University of Chinese Medicine. The dried herbs were immersed in purified water (1, 10, w/v) for 30 min, decocted for a further 30 min, and filtered to yield the first extract. The herbs were decocted again with eight times the initial volume of water for 20 min to produce the second extract (Yang et al., 2021). The mixture was filtered, and the two resulting extracts were combined. The combined extracts were finally filtered, aliquoted, and stored at 4 °C in the dark. Alendronate Sodium Tablets (ALN, WO24849, Savio Industrial S.r. L., Italy) were pulverized and suspended in physiological saline at a concentration of 0.16 mg/mL for delivery.

### 2.5 Animal husbandry and grouping

The Committee for the Protection of Animal Welfare at Changchun University of Chinese Medicine conducted a review and granted approval for all animal research (Approval No. SYXK (Ji) 2023–0015). This research was carried out in strict compliance with national standards regulating animal experimentation. The study encompassed 48 specific-pathogen-free (SPF) non-pregnant female rats (3 months old, 220 ± 20 g), all of which had unrestricted access to standard rodent chow and water. Following a week of acclimatization, Sprague Dawley (SD) rats were divided into a Sham group (*n* = 8) and an OVX group (*n* = 40). Following anesthesia induced by intraperitoneal injection of 2% sodium pentobarbital (20 mg/mL), bilateral ovariectomy was performed in the OVX group, while the Sham group underwent a sham operation involving only the removal of the peri-ovarian fat tissue. Ceftriaxone (20 mg/kg) was injected intraperitoneally 30 min post-surgery to prevent infection. Gavage administration was initiated 1 week subsequent to post-OVX to evaluate its efficacy in reversing established bone loss. Saline was administered to the Sham and OVX groups at a dosage of 2 mL/kg/day. The SJP-H group was administered SJP at a dosage of 11.4 g/kg/day, the SJP-M group at 5.7 g/kg/day, and the SJP-L group at 2.85 g/kg/day for a duration of 12 weeks, as determined by the body surface area method for rats (Nair and Jacob, 2016), and the conversion factor (Km) was set to 7.0. The ALN group received ALN at a dosage of 0.16 mg/mL by gavage administration (Jiang et al., 2025). The animals were weighed weekly, and gavage doses were adjusted accordingly to achieve precise dosing.

### 2.6 Collection of different specimens

Twenty-four hours following the final intragastric administration, SD rats were sedated with sodium pentobarbital. Following blood collection, serum was separated via centrifugation for utilization in subsequent ELISA assays. Colon tissue and fecal specimens were obtained for the study of gut microbiota and metabolites. The left femur was removed for histological examination, while the right femur was utilized for micro-computed tomography (μCT) imaging to evaluate bone morphology.

### 2.7 Hematoxylin–eosin (H&E) staining

The femur samples were fixed in a 4% paraformaldehyde solution for a period of 24 h. Subsequently, they were rinsed with a 10% EDTA solution and subjected to decalcification at room temperature for a duration of 2 months. Following this, the decalcified bone tissue was dehydrated, embedded in paraffin, and sectioned into 5-μm slices. H&E staining was conducted to evaluate bone morphology. Trabecular bone area fraction (%), defined as the ratio of trabecular bone area to total bone area, was quantified using ImageJ (v1.8.0).

### 2.8 Micro-computed tomography (μCT)

The specimens were examined with a μCT machine (Quantum GX2, PerkinElmer, United States). The scanning parameters were a tube voltage of 90 kV, a tube current of 88 μA, and a voxel size of 36 × 36 μm. Data collection was executed with Cruiser, picture reconstruction was carried out in Recon, and analysis was performed utilizing Avatar. The structural properties of these forms of bone were quantified (Jiang et al., 2025).

### 2.9 Enzyme-linked immunosorbent assay (ELISA)

Serum samples were thawed at 4 °C and examined for Procollagen Type I N-terminal Propeptide (PINP), Bone Morphogenetic Protein 2 (BMP2), Tartrate-Resistant Acid Phosphatase 5b (TRACP5b), TNF-*α*, interleukin-1 beta (IL-1β), and interleukin-10 (IL-10). All assays were performed using ELISA kits, following the protocols specified by the manufacturer. The ELISA kits were procured from DO Biological (Nanjing Jiancheng Bioengineering Institute, China).

### 2.10 16S rRNA microbial community analysis

The genomic DNA was extracted using the MagPure Soil DNA LQ Kit (Magan). To evaluate the concentration and quality of the DNA, NanoDrop 2000 spectrophotometry and agarose gel electrophoresis were employed. The purified DNA was subsequently stored at −20 °C. To conduct an analysis of the composition of the bacterial community, fragments of the bacterial 16S rRNA gene were amplified through PCR using barcoded primers and Takara Ex Taq polymerase, with the universal primers 343F and 798R targeting the V3–V4 hypervariable regions (Liu J. et al., 2023). Quantification of the generated amplicons was carried out using the Qubit dsDNA Assay Kit (Li et al., 2022). Qiime2 (November 2020) utilized the DADA2 algorithm for the processes of filtering, denoising, merging, and chimera removal, ultimately producing an abundance table of Amplicon Sequence Variants (ASVs) and their corresponding representative sequences. To classify these sequences, the q2-feature-classifier plugin was applied, and the sequences were compared with the SILVA database (version 138) (Bolyen et al., 2019). Microbiome profiling used five samples per group to ensure biological variability and robust multivariate analysis (Chijiiwa et al., 2020; McAdams et al., 2026).

### 2.11 Non-targeted metabolomics analyses of the feces

Metabolites were isolated from murine excrement utilizing liquid nitrogen and methanol, with quality control samples guaranteeing data integrity. The Vanquish UHPLC platform, an Orbitrap Q Exactive HF-X mass spectrometer, and a Hypersil Gold column were employed in this experiment. Subsequently, metabolite pathway enrichment analysis was performed with MetaboAnalyst 6.0 and the KEGG database. Metabolite identification was outsourced to Shanghai OE Biotech Co., Ltd. Five samples per group were utilized for metabolomic profiling to guarantee robust multivariate analysis and capture biological variability (Zheng et al., 2016).

### 2.12 Traceability analysis of differential metabolites

The MetOrigin platform^10^ was employed to conduct a retrospective traceability study of divergent metabolites, enabling origin, function, and Sankey network analyses (Qiu et al., 2025).

### 2.13 Molecular docking

Molecular docking analyses of SJP-activating chemicals aimed at Cyclooxygenase-2 (COX2, PDB ID: 1CX2) and Prostaglandin E Synthase (PTGES, PDB ID: 1Z9H), pivotal enzymes in AA metabolism, were conducted utilizing protein structures sourced from the RCSB Protein Data Bank^11^. Molecular docking simulations were carried out utilizing AutoDock Tools version 1.5.7, and the outcomes were analyzed via Discovery Studio 2019 Client and PyMOL.

### 2.14 Molecular dynamics simulation

The compounds that exhibited the highest binding affinities for PTGES and COX2 in molecular docking were selected for molecular dynamics (MD) simulations. The preparation of protein-ligand complexes was carried out using GROMACS. Subsequently, the complexes were solvated in a water-filled box and then neutralized through the addition of ions. Upon completion of energy minimization and equilibration under physiological conditions, a 100-nanosecond production run was conducted. The structural stability and dynamic flexibility of the complexes were evaluated through the examination of several features, including the root mean square deviation (RMSD), root mean square fluctuation (RMSF), and radius of gyration (Rg). Furthermore, solvent-accessible surface area (SASA) was computed to evaluate binding interactions, with a decrease in SASA signifying effective ligand binding within the active sites. Hydrogen bond interactions were monitored to evaluate complex stability of the complexes. Additionally, binding free energies were calculated to determine the relative binding affinities (Jiang et al., 2025).

### 2.15 Western blot

Following transfer of the proteins onto a PVDF membrane, the membrane was incubated overnight at 4 °C with primary antibodies including PI3K (20584-1-AP), AKT (10176-2-AP), p-AKT (66444-1-Ig), GAPDH (60004-1-Ig), ZO-1 (21773-1-AP), and Occludin (27260-1-AP) from Proteintech, as well as p-PI3K (bs-6417R) from Bioss. Subsequently, the membrane was incubated with an HRP-conjugated secondary antibody for 1 h. Protein bands were visualized by ECL and quantified using ImageJ (v1.8.0).

### 2.16 Immunohistological analysis

The femur tissue sections were fixed in 0.1 M Tris-buffered saline for a duration of 10 min. Following a 1-h blocking step in PBS supplemented with 10% normal goat serum, the sections were incubated overnight at 4 °C with primary antibodies from Servicebio targeting OCN (GB11233), RUNX2 (GB115631), and EP4 (GB111814). Following the washing process, the samples were treated with a secondary antibody conjugated to horseradish peroxidase (GB21301, Servicebio, China) for 1 h at room temperature. Immunoreactivity was identified utilizing 3,3′-diaminobenzidine (DAB) as the chromogenic substrate. The quantification of immunopositive signals was conducted employing ImageJ software (version 1.8.0), evaluating six randomly selected high-power fields (HPF) per sample to assess marker expression levels.

### 2.17 Data processing and analysis

Data are presented as mean ± standard deviation. Statistical analyses were conducted using GraphPad Prism software (version 10). All data, with the exception of weight measurements analyzed by repeated measures ANOVA, were analyzed using one-way ANOVA. Results were deemed statistically significant at *p < 0.05, **p < 0.01, and ***p < 0.001, with data that were not applicable explicitly marked as N. A.

## 3 Results

### 3.1 SJP alleviates bone loss and histopathological damage

**Figure 1:**
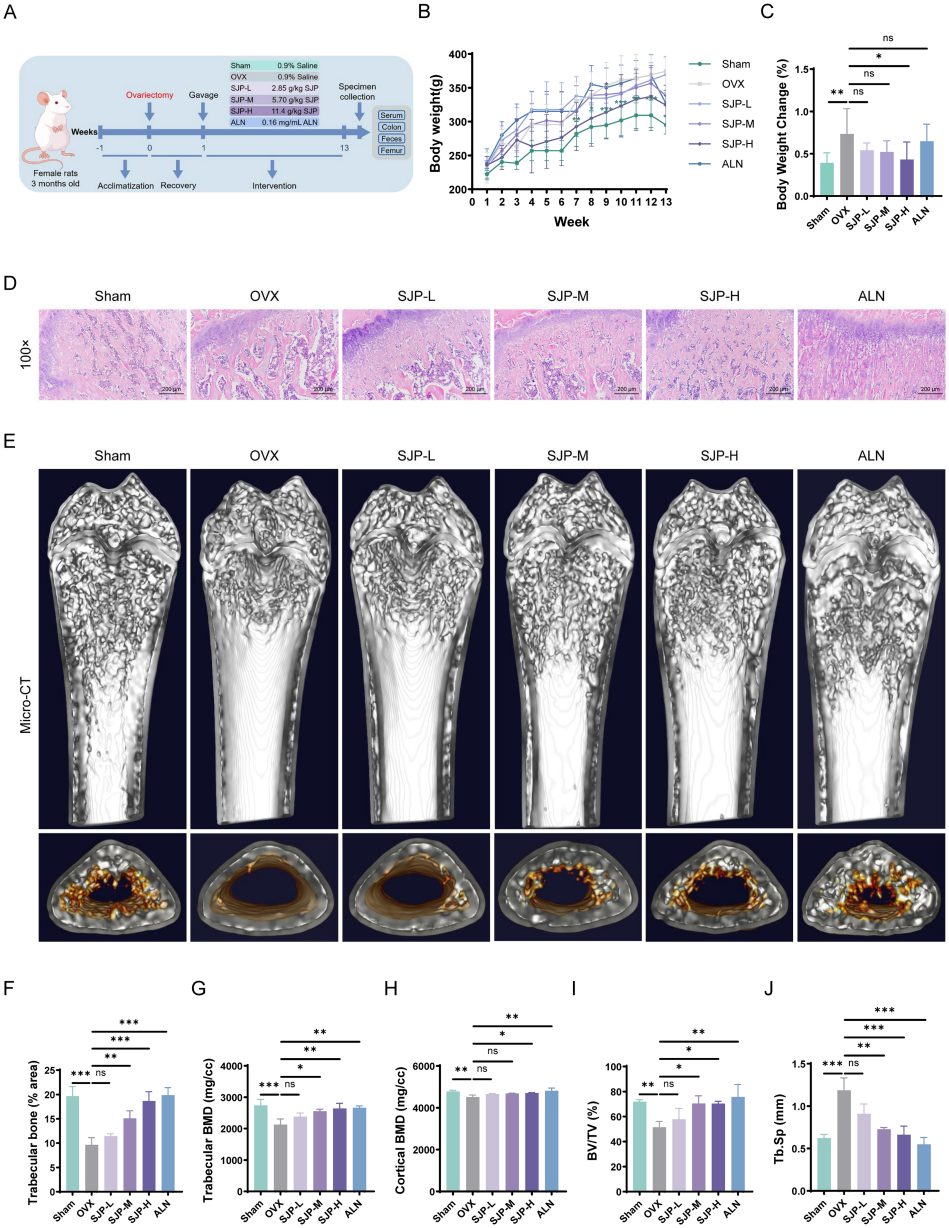
Effects of SJP treatment on OVX-induced weight gain, uterine atrophy, and bone loss in rats. **(A)** Diagram depicting the experimental setup. **(B)** Weekly body weight changes of rats during the treatment period. **(C)** Body weight changes at the start and end of the experiment. **(D)** H&E staining (scale bar = 200 μm). **(E)** Micro-CT images of the distal femur from each group of rats. **(F)** Trabecular area in femur was analyzed by Image J program. **(G–J)** Micro-CT analysis provided the following parameters for the distal femur: **(G)** Trabecular BMD, **(H)** Cortical BMD, **(I)** BV/TV, and **(J)** Tb. Sp. Data are shown as mean ± SEM (*n* = 3). *p < 0.05, **p < 0.01, ***p < 0.001.

13-Week Experimental Protocol for PMOP Modeling and Gastric Administration Treatment (Figure 1A). Aberrant weight gain is a hallmark metabolic feature associated with estrogen deficiency in the OVX rat model (Chalvon-Demersay et al., 2017). To evaluate the efficacy of SJP in regulating this metabolic alteration, we monitored the weekly body weight trajectories of the rats. Over the 13-week trial, SJP-H treatment reduced OVX-induced aberrant weight gain, with effects becoming apparent by week 3 and reaching statistical significance by week 9 (*p* < 0.05) (Figure 1B). SJP-M and SJP-L also suppressed OVX-related weight gain, with noticeable effects starting from week 8. Analysis of the percentage change in final body weight revealed that the weight gain in the OVX group was significantly greater than that in the sham group (*p* < 0.01). In contrast, ALN treatment was ineffective in counteracting the OVX-induced aberrant weight gain (Figure 1C). Collectively, these findings indicate that SJP mitigates OVX-induced aberrant weight gain.

To assess the efficacy of SJP against osteoporosis, we conducted histological and micro-architectural analyses of bone tissues employing H&E staining and μCT imaging. The histological analysis revealed a significant deterioration of the trabecular microstructure in the OVX group compared to the Sham group, manifested as trabecular attenuation, architectural disorganization, and enlarged marrow cavities with frequent vacuole-like structures (Figure 1D). In contrast, treatment with ALN and SJP, particularly at the SJP-H/M dosage, resulted in notable improvements in trabecular number and continuity, along with a reduction in bone marrow adiposity. These histological findings were further supported by 3D μCT imaging. Representative sagittal and transverse sections of the femur demonstrated that all SJP-treated groups exhibited improved trabecular architecture and increased cortical thickness relative to the OVX model (Figure 1E). Subsequently, H&E quantitative analysis indicated that the trabecular bone area was significantly lower in the OVX group compared to the Sham group (*p* < 0.001), and it was significantly higher subsequent to treatment with SJP (*p* < 0.01) (Figure 1F). μCT measurements indicated that, compared to the Sham group, the trabecular BMD, cortical BMD, and BV/TV in the OVX group were markedly decreased (*p* < 0.01), while the Tb. Sp was significantly increased (*p* < 0.01). Consistent with the imaging findings, SJP treatment dose-dependently elevated trabecular BMD, cortical BMD, and BV/TV (*p* < 0.05), while significantly reducing Tb. Sp (*p* < 0.001) (Figures 1G–J). Collectively, these results demonstrate that SJP effectively normalized cortical and cancellous bone density and restored the trabecular microarchitecture.

### 3.2 SJP enhances osteoblast activity and modulates systemic inflammation

**Figure 2:**
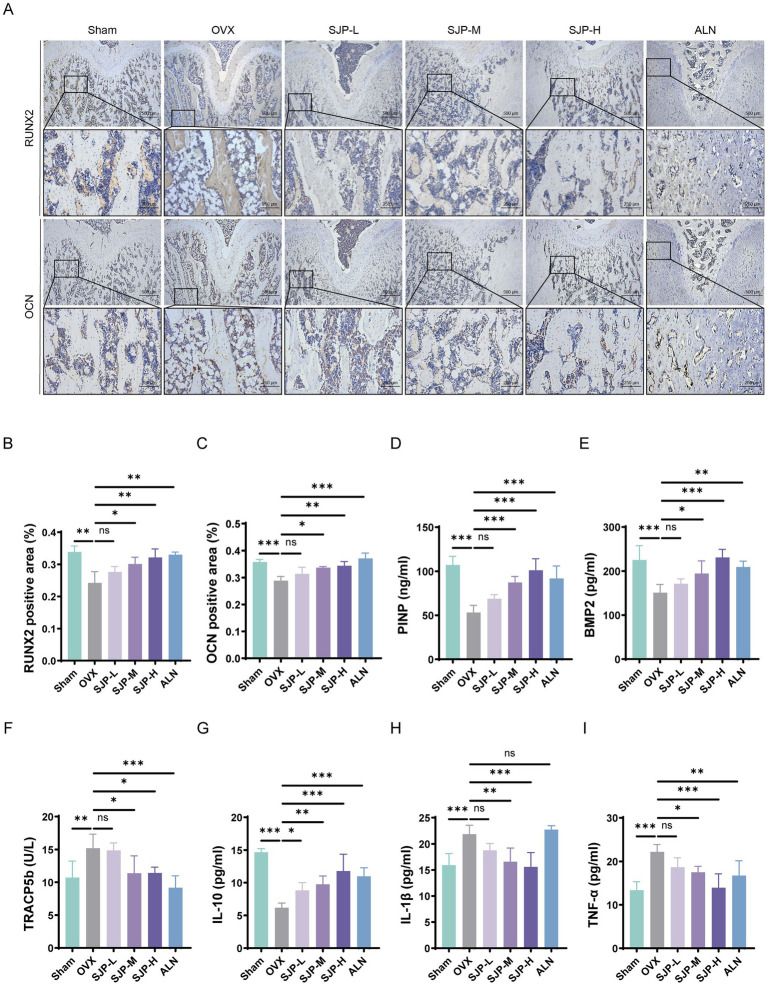
SJP treatment enhances osteogenesis. **(A)** Immunohistochemical staining showing the expression of RUNX2 and OCN in the femur. Quantitative analysis of RUNX2 **(B)** and OCN **(C)** expression in the femur via immunohistochemistry. Serum levels of bone markers were assessed utilizing ELISA, including PINP **(D)**, and BMP2 **(E)**, and TRACP5b **(F)**. Additionally, serum levels of inflammatory markers, IL-10 **(G)**, IL-1β **(H)**, and TNF-α **(I)**, were measured utilizing ELISA. Data are shown as mean ± SEM (*n* = 3). *p < 0.05, **p < 0.01, ***p < 0.001.

To investigate the influence of SJP on osteoblast function, we measured the levels of key osteoblast markers RUNX2 and OCN in femoral samples using IHC staining. Compared to the OVX group, the SJP-H/M treatment group exhibited stronger immunostaining signals for both RUNX2 and OCN proteins (Figure 2A). The results indicated that the expression levels of RUNX2 and OCN in the SJP-H/M group were significantly higher than those in the OVX group (*p* < 0.01) (Figures 2B,C). Having established the local osteogenic effects, we explored their correlation with systemic bone metabolism. The ELISA results indicated that, compared to the model group, SJP treatment significantly elevated serum levels of PINP and BMP2, which are markers of bone formation (*p* < 0.01), and reduced the levels of TRACP5b, a marker of bone resorption (*p* < 0.05) (Figures 2D–F). Given the well-documented association between chronic inflammation and PMOP, we subsequently assessed the systemic anti-inflammatory properties of SJP. The ELISA results revealed that SJP treatment modulates key inflammatory mediators by increasing IL-10 levels while suppressing IL-1β and TNF-α, indicating its ability to ameliorate the PMOP-associated systemic inflammatory microenvironment (Figures 2G–I). Collectively, SJP acted through a coordinated regulation of local osteoblast activity, systemic bone anabolism, and the inflammatory microenvironment.

### 3.3 SJP modulates the structure and function of gut microbiota in OVX rats

**Figure 3:**
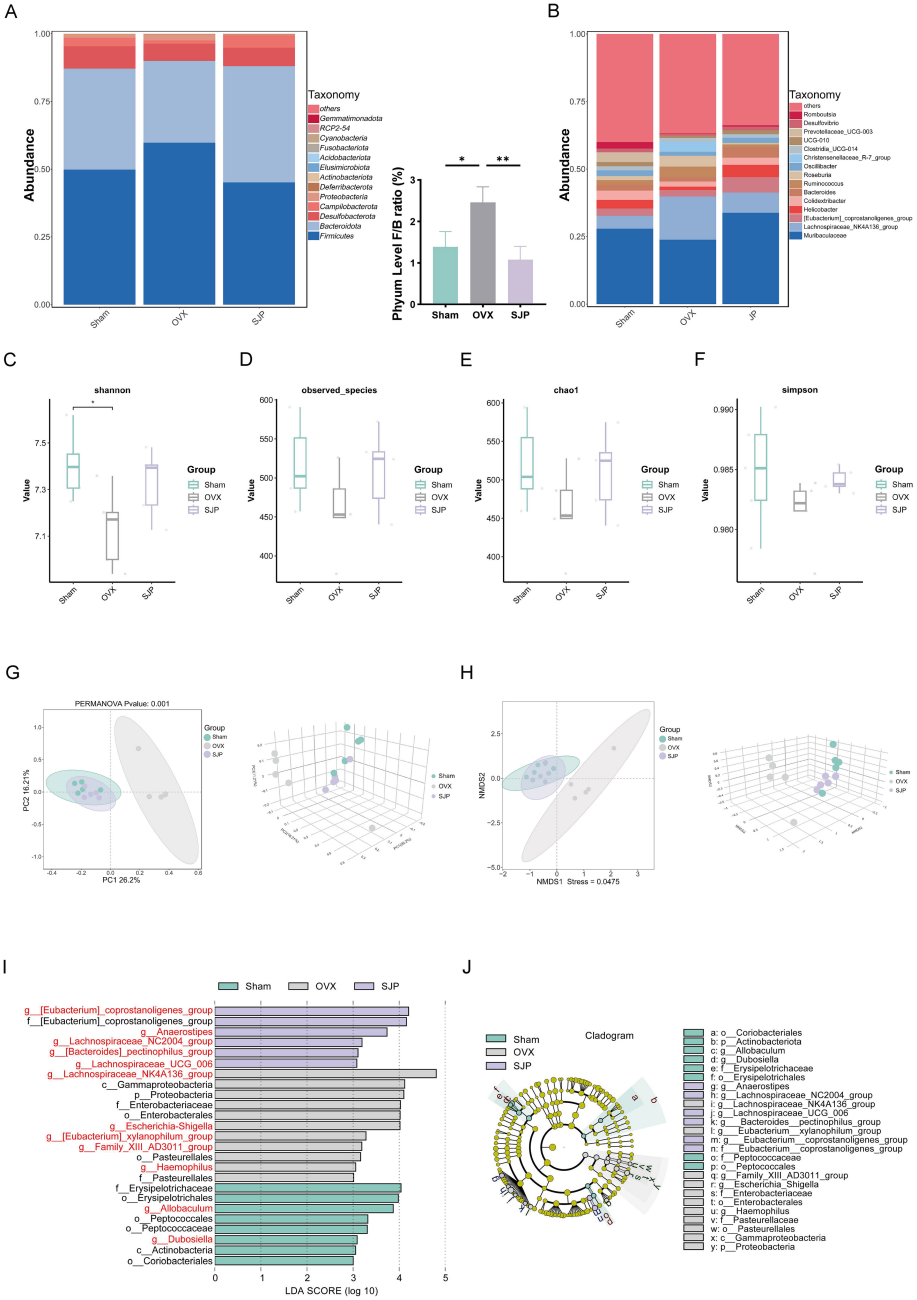
SJP mitigates OVX-induced gut microbiota dysbiosis. **(A)** Bacterial community structure at the phylum level. **(B)** Archaeal community structure at the genus level. **(C)** Shannon species. **(D)** Observed index. **(E)** Chao1 index. **(F)** Simpson index. **(G,H)** Beta diversity analysis, including PCoA **(G)** and NMDS **(H)**. **(I,J)** LEfSe analysis comparing microbial variation at the genus level across three groups. LEfSe branch diagrams highlight differentially abundant taxa (*p* < 0.05). LDA scores calculated from LEfSe for each taxon show significant differences between groups, with only taxa exhibiting LDA scores >3 displayed. Data are shown as mean ± SEM (*n* = 5). *p < 0.05, **p < 0.01.

To analyze the influence of SJP on the gut microbiota in PMOP rats, fecal samples were subjected to 16S rRNA sequencing. Analysis of the microbial composition at the phylum level revealed that Firmicutes were elevated in the OVX group, while SJP-H treatment significantly reversed the OVX-induced increase in the *Firmicutes/Bacteroidetes* (F/B) ratio, restoring a microbial profile dominated by *Bacteroidetes* (Figure 3A). At the genus level, *Muribaculaceae*, *[Eubacterium]_coprostanoligenes_group*, *Helicobacter*, and *Colidextribacter* exhibited greater prevalence in the Sham and SJP-H groups, whereas *Lachnospiraceae_NK4A136_group* was more abundant in the OVX group (Figure 3B and Supplementary Figures S1A–E). Alpha diversity analysis was performed to evaluate microbial diversity. The rank-abundance curve showed considerable richness and evenness, with the species accumulation curve reaching a saturation plateau, confirming adequate sequencing depth (Supplementary Figures S1F,G). Compared with the OVX group, both Sham and SJP-H groups showed higher alpha-diversity across all indices. The Shannon index was significantly higher in Sham and SJP-H versus OVX (*p* < 0.05); Observed species, Simpson, and Chao1 also trended higher, though not significantly, demonstrating that SJP-H treatment effectively restored gut microbiota richness and diversity in OVX rats (Figures 3C–F). Principal Coordinate Analysis (PCoA) and Non-metric Multidimensional Scaling (NMDS) of beta diversity indicate that the OVX group is distinctly separated from the other two groups, while the data points of the SJP-H group are closely clustered with those of the Sham group (Figures 3G,H). These results indicate that ovariectomy significantly altered gut microbiota architecture, while SJP-H treatment effectively reversed these OVX-induced structural alterations. LEfSe analysis (LDA score > 3, *p* < 0.05) revealed significant differences in 12 bacterial taxa at the genus level among the three groups (Figures 3I,J). The Sham group exhibited an abundance of *Allobaculum* and *Dubosiella*. In contrast, the OVX group was characterized by five specific genera, including *Lachnospiraceae_NK4A136_group* and *Escherichia_Shigella*. Meanwhile, the SJP-H group demonstrated an enrichment of five genera, such as the *[Eubacterium]_coprostanoligenes_group* and *Anaerostipes*. Collectively, these findings suggest that SJP-H effectively altered the gut microbiota composition in OVX-induced PMOP rats, thereby mitigating microbial dysbiosis.

### 3.4 SJP modulates metabolic disturbances induced by OVX

**Figure 4:**
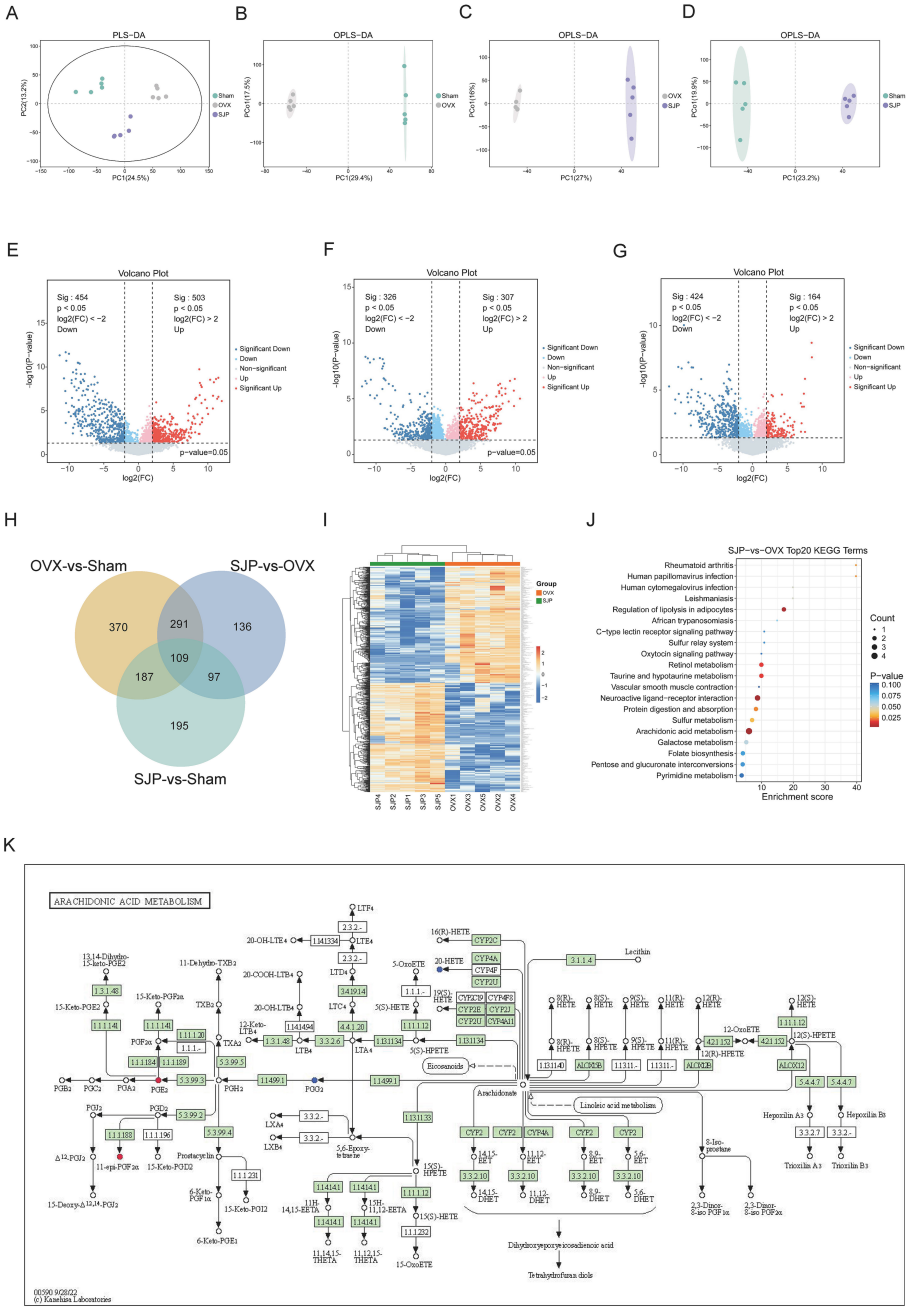
Untargeted metabolomic analysis of fecal samples. **(A)** PLS-DA of LC–MS data comparing OVX, SJP, and Sham groups. **(B)** OPLS-DA of LC–MS data comparing OVX and Sham groups. **(C)** OPLS-DA of LC–MS data comparing SJP and OVX groups. **(D)** OPLS-DA of LC–MS data comparing SJP and Sham groups. **(E)** Volcano plot of differential metabolites between OVX and Sham groups. **(F)** Volcano plot of differential metabolites between SJP and OVX groups. **(G)** Volcano plot of differential metabolites between SJP and Sham groups. In the volcano plots, each point represents a metabolite. Red points indicate up-regulated metabolites with statistical significance, while blue points represent down-regulated metabolites with statistical significance. Gray points denote metabolites with no significant change. **(H)** Venn diagram illustrating the differentially regulated metabolites across the three groups. **(I)** Heatmap displaying the metabolite differences among the three groups. **(J)** KEGG pathway enrichment analysis of differentially expressed metabolites, highlighting the top 20 enriched pathways. **(K)** KEGG pathway map for AA metabolism. Data are shown as mean ± SEM (*n* = 5).

To characterize the modulatory effect of SJP on overall metabolism, we collected intestinal contents from rats and conducted a metabolomic analysis. The PLS-DA analysis demonstrated a distinct separation among the fecal metabolic profiles of the three rat groups (Figure 4A). OPLS-DA analysis revealed distinct metabolic profiles among the Sham, OVX, and SJP groups (Supplementary Figure S2A), with significant separations observed in all pairwise comparisons. The clear distinction between the OVX group and the Sham group (*R*^2^*X* = 0.539, *R*^2^*Y* = 0.999) demonstrated that ovariectomy induced substantial metabolic alterations (Figure 4B and Supplementary Figure S2B). Furthermore, SJP treatment significantly modulated systemic metabolism, as evidenced by its unique metabolic profile that diverged from both the OVX group (*R*^2^*X* = 0.611, *R*^2^*Y* = 0.999, *Q*^2^ = 0.877) and the Sham group (*R*^2^*X* = 0.597, *R*^2^*Y* = 0.997, *Q*^2^ = 0.905) (Figures 4C,D and Supplementary Figures S2C,D). The robustness of these models was validated through permutation tests (*n* = 200), with all permuted *Q*^2^ values falling below their original counterparts and negative *Q*^2^ intercepts, indicating the absence of overfitting. By employing thresholds of VIP > 1, a significance level of *p* < 0.05, and fold change (FC) ≥ 2.0 or ≤0.25, we subsequently screened for differentially expressed metabolites and visualized the results in volcano plots. Comparative analysis between the OVX group and the Sham group revealed 957 differential metabolites, including 503 upregulated and 454 downregulated (Figure 4E). SJP intervention resulted in 633 differentially expressed metabolites compared to the OVX group, with 307 upregulated and 326 downregulated (Figure 4F). Comparison with the Sham group revealed 588 differential metabolites, consisting of 164 upregulated and 424 downregulated (Figure 4G). Among the 400 metabolites that overlapped between the OVX-Sham and SJP-OVX comparisons, we excluded those exhibiting concordant change directions in both the SJP and OVX groups, resulting in 384 metabolites whose alterations were effectively reversed by SJP treatment (Figures 4H,I). The KEGG pathway enrichment analysis, based on these reversed metabolites, identified 54 significantly enriched pathways, with the top 20 ranked by *p*-value presented in Figure 4J. Screening of the HMDB database identified 17 key metabolites, including PGE2, which is involved in arachidonic acid metabolism and rheumatoid arthritis; 20-HETE acid, associated with vascular smooth muscle contraction; and glutaric acid, linked to fatty acid degradation and lysine metabolism (Figure 4K; Table 1; Supplementary Figures S2E–G). Overall, these results demonstrate that SJP ameliorated OVX-induced systemic metabolic dysregulation by reversing OVX-induced metabolite abnormalities and regulating critical pathways such as arachidonic acid metabolism.

**Table 1:** 17 differential metabolites of three groups.

Metabolites	Formula	FoldChange (SJP-vs-OVX)	log2FoldChange (SJP-vs-OVX)	VIP (SJP-vs-OVX)	Regulation (OVX-vs-Sham)	Regulation (SJP-vs-OVX)
Tyramine	C_8_H_11_NO	0.030049	−5.05653	2.701036199	Up	Down
All-*trans*-5,6-Epoxyretinoic acid	C_20_H_28_O_3_	36.13259	5.175229	2.70987336	Down	Up
Prephenate	C_10_H_10_O_6_	0.16233	−2.623	1.869845054	Up	Down
Deoxycytidine	C_9_H_13_N_3_O_4_	0.175932	−2.50691	1.809767309	Up	Down
All-*trans*-4-Oxoretinoic acid	C_20_H_26_O_3_	11.69431	3.547735	2.18571042	Down	Up
11-Epi-PGF2a	C_20_H_34_O_5_	4.340718	2.117934	1.682745537	Down	Up
20-Hydroxyeicosatetraenoic acid	C_20_H_26_D_6_O_3_	0.098615	−3.34206	2.071559085	Up	Down
Glycerol	C_3_H_8_O_3_	0.213769	−2.22587	1.690275915	Up	Down
Prostaglandin E2	C_20_H_32_O_5_	8.592423	3.103065	2.000298873	Down	Up
2,4-dihydroxy-2-heptenedioc acid	C_7_H_10_O_6_	0.214902	−2.21825	1.652629411	Up	Down
L-galactono-1,4-lactone	C_6_H_10_O_6_	0.234076	−2.09495	1.589589482	Up	Down
Glutaric acid	C_5_H_8_O_4_	0.196451	−2.34776	1.681433224	Up	Down
Nicotinuric acid	C_8_H_8_N_2_O_3_	0.121819	−3.03718	1.890424123	Up	Down
Pectic acid	C_6_H_10_O_7_	0.162244	−2.62377	1.749928613	Up	Down
L-Dihydroorotic acid	C_5_H_6_N_2_O_4_	0.178249	−2.48803	1.706184633	Up	Down
Sepiapterin	C_9_H_11_N_5_O_3_	0.155136	−2.6884	1.729659604	Up	Down
3-Hydroxy-2-methylpyridine-4,5-dicarboxylate	C_8_H_7_NO_5_	0.099287	−3.33225	1.884275615	Up	Down

### 3.5 Metabolite profiling and source-based analysis of metabolic functions

**Figure 5:**
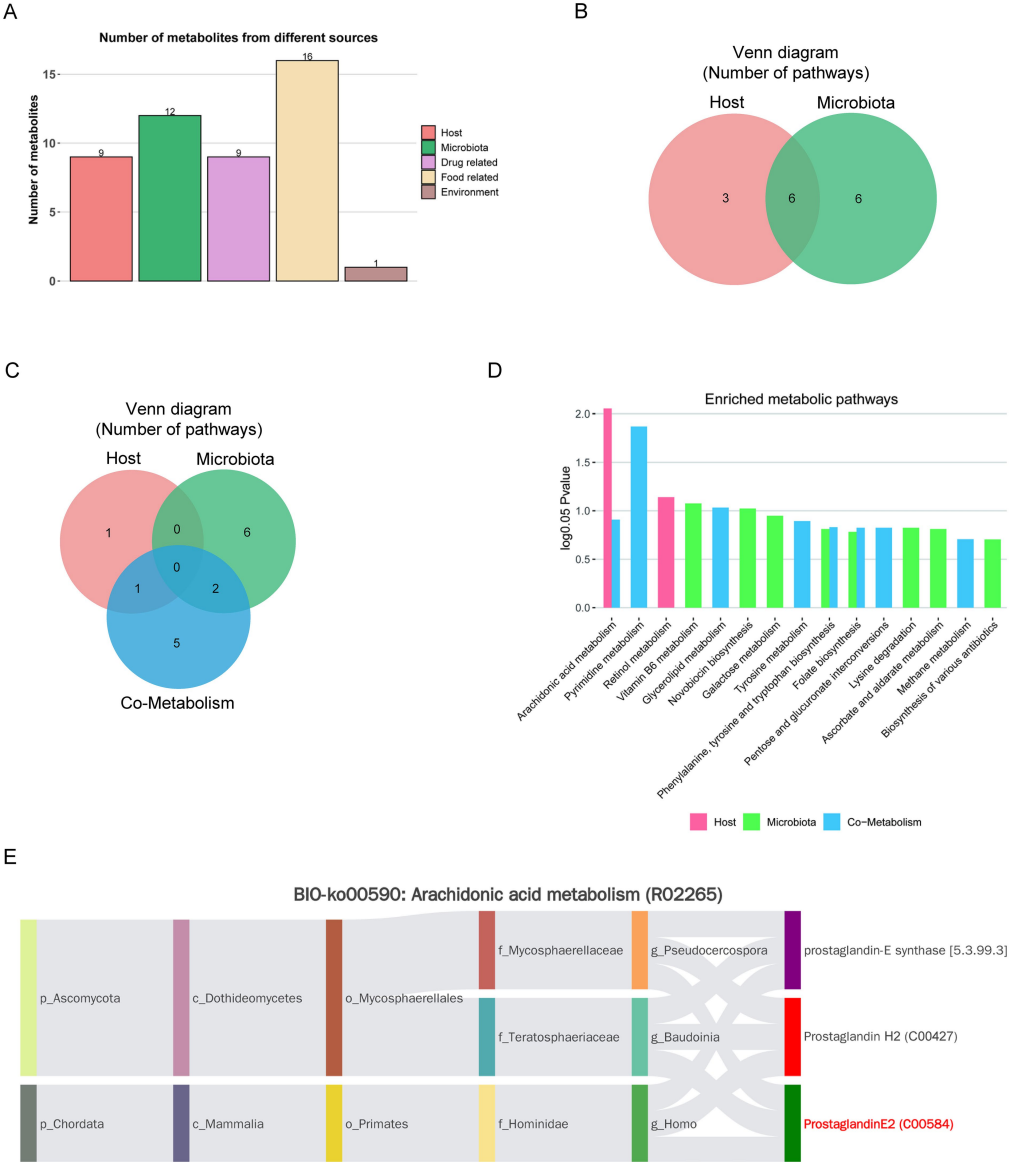
MetOrigin tracing analysis of differential metabolites. **(A)** Bar plot showing the distribution of metabolites across different sources. **(B)** Venn diagram comparing the number of metabolites in the human and bacterial communities. **(C)** Venn diagram comparing the metabolic pathways in the human and bacterial communities. **(D)** Bar plot illustrating the enriched metabolic pathways in both communities. **(E)** BIO-Sankey network diagram of AA metabolism (R02265). Dark red bars represent metabolic substrates, dark green bars indicate metabolic products, and purple bars represent metabolic enzymes.

The metabolite-microbiome origin analysis performed by MetOrigin revealed a diverse range of metabolic origins in the feces of OVX rats (Yu G. et al., 2024). We identified six co-metabolites that originated from both the microbiota and the host, three metabolites exclusive to the host, and six metabolites unique to the microbiota. Furthermore, we detected 16 metabolites associated with food, 9 linked to drugs, and 1 related to the environment (Figures 5A,B). Functional analysis indicated that two metabolic pathways were associated with host-specific data, eight with microbiota-specific data, and eight with co-metabolism data (Figure 5C). A significant metabolic pathway associated with OVX (log_0.05_ *P* > 1) is AA metabolism, which exhibits the highest level of significance (log_0.05_ *p* > 1.5) and primarily encompasses the metabolic processes shared by both the host and the microbial community (Figure 5D). Additionally, Bio-Sankey networks were employed to investigate the statistical relationships and biological associations between metabolites and microbiota (Figure 5E and Supplementary Figures S2E,F). Within the arachidonic acid (AA) metabolic pathway, three specific metabolites are involved: prostaglandin F2alpha, (5Z,13E)-11alpha-Hydroxy-9,15-dioxoprost-5,13-dienoate ALA, and PGE2. These metabolites participate in three distinct reactions, specifically R02581, R02580, and R02265.

### 3.6 Active compounds and target networks in the treatment of PMOP by SJP

**Figure 6:**
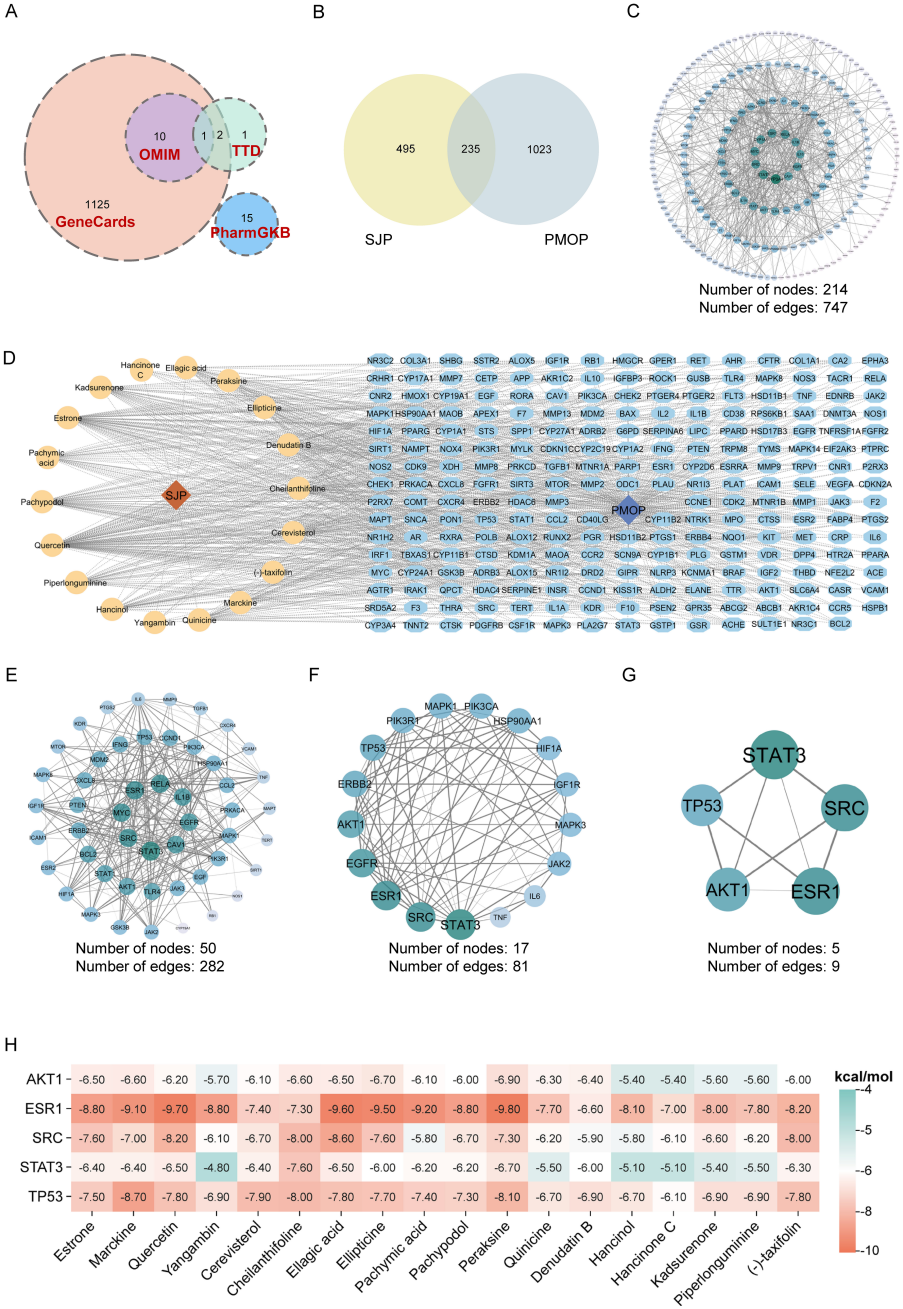
Analysis of PMOP-related targets for SJP treatment. **(A)** PMOP-related targets retrieved from various databases, including GeneCards (*n* = 1,125), OMIM (*n* = 11), PharmGKB (*n* = 15), and TTD (*n* = 4). **(B)** Venn diagram illustrating the overlap between SJP-related targets and PMOP-related targets. **(C)** Protein–protein interaction (PPI) network of overlapping targets, consisting of 214 nodes and 747 edges. **(D)** Network diagram depicting the active ingredients of SJP, predicted targets, and PMOP-related diseases. **(E–G)** Identification of core targets utilizing CytoNCA, with darker colors indicating higher scores. **(H)** Cluster heatmap of molecular docking results between hub genes and active compounds.

To systematically identify the effective components and key targets of SJP in the treatment of PMOP, we employed a method that integrates network pharmacology and molecular docking. Screening of public databases, including TCMSP, Herb, and BATMAN-TCM, identified 492 compounds present in SJP. Based on criteria such as gastrointestinal absorption and oral bioavailability, we screened these 492 compounds using the SwissADME platform and TCMSP database, yielding 18 potential bioactive compounds (Table 2). The potential targets of these 18 compounds were predicted by cross-referencing them with the TCMSP and Swiss Target Prediction databases, resulting in 730 unique targets after removing duplicates. Concurrently, PMOP-related targets were retrieved from several disease databases and processed to eliminate redundancy, yielding 1,258 unique targets (Figure 6A). The overlapping targets of the SJP-compound and PMOP-related datasets allowed us to identify 235 identical entities (Figure 6B). Subsequently, a PPI network was constructed for these 235 common targets. This network was refined by retaining only interactions with a high confidence score (combined score ≥ 0.900) and removing isolated nodes, resulting in a core PPI network of 214 targets (Figure 6C and Supplementary Figure S3). The Cytoscape software illustrated the associations between the 18 SJP compounds and genes related to PMOP through gene visualization (Figure 6D). Utilizing established centrality criteria, topological analysis with CytoNCA identified five hub genes within this core network, specifically STAT3, TP53, AKT1, ESR1, and SRC (Figures 6E–G). To validate the interactions suggested by the network pharmacology results, molecular docking simulations were conducted with key active compounds such as estrone and quercetin in relation to the core target proteins. The docking results demonstrated strong binding affinities, with binding energies lower than −6.0 kcal/mol, thereby reinforcing the reliability of our network-based predictions (Figure 6H and Supplementary Figures S4A–E). In summary, this study identified 18 potential active components in SJP and pinpointed STAT3, TP53, AKT1, ESR1, and SRC as its core targets against PMOP. This substantiates the network-based predictions through molecular docking and provides a solid foundation for its multi-target therapeutic mechanism.

**Table 2:** The 18 active compounds in SJP.

No.	Compounds	Formula	CAS	OB (%)	DL	GI absorption
1	Estrone	C_18_H_22_O_2_	53–16-7	53.56	0.32	High
2	Marckine	C_29_H_37_N_3_O_3_	2,632-29-3	37.05	0.69	High
3	Quercetin	C_15_H_10_O_7_	117–39-5	46.43	0.28	High
4	Yangambin	C_24_H_30_O_8_	13,060–14-5	57.53	0.81	High
5	Cerevisterol	C_28_H_46_O_3_	516–37-0	39.52	0.77	High
6	Cheilanthifoline	C_19_H_19_NO_4_	483–44-3	46.51	0.72	High
7	Ellagic acid	C_14_H_6_O_8_	476–66-4	43.06	0.43	High
8	Ellipticine	C_17_H_14_N_2_	519–23-3	30.82	0.28	High
9	Pachymic acid	C_33_H_52_O_5_	29,070–92-6	33.63	0.81	High
10	Pachypodol	C_18_H_16_O_7_	33,708–72-4	75.06	0.4	High
11	Peraksine	C_19_H_22_N_2_O_2_	15,527–80-7	82.58	0.78	High
12	Quinicine	C_20_H_24_N_2_O_2_	84–55-9	75.44	0.33	High
13	Denudatin B	C_21_H_24_O_5_	87,402–88-8	61.47	0.38	High
14	Hancinol	C_22_H_28_O_5_	108,864–50-2	64.01	0.37	High
15	Hancinone C	C_23_H_28_O_6_	111,843–10-8	59.05	0.39	High
16	Kadsurenone	C_21_H_24_O_5_	95,851–37-9	54.72	0.38	High
17	Piperlonguminine	C_16_H_19_NO_3_	5950/12/9	30.71	0.18	High
18	(−)-taxifolin	C_15_H_12_O_7_	111,003–33-9	60.51	0.27	High

### 3.7 Analysis of essential pathways in SJP for the treatment of PMOP

**Figure 7:**
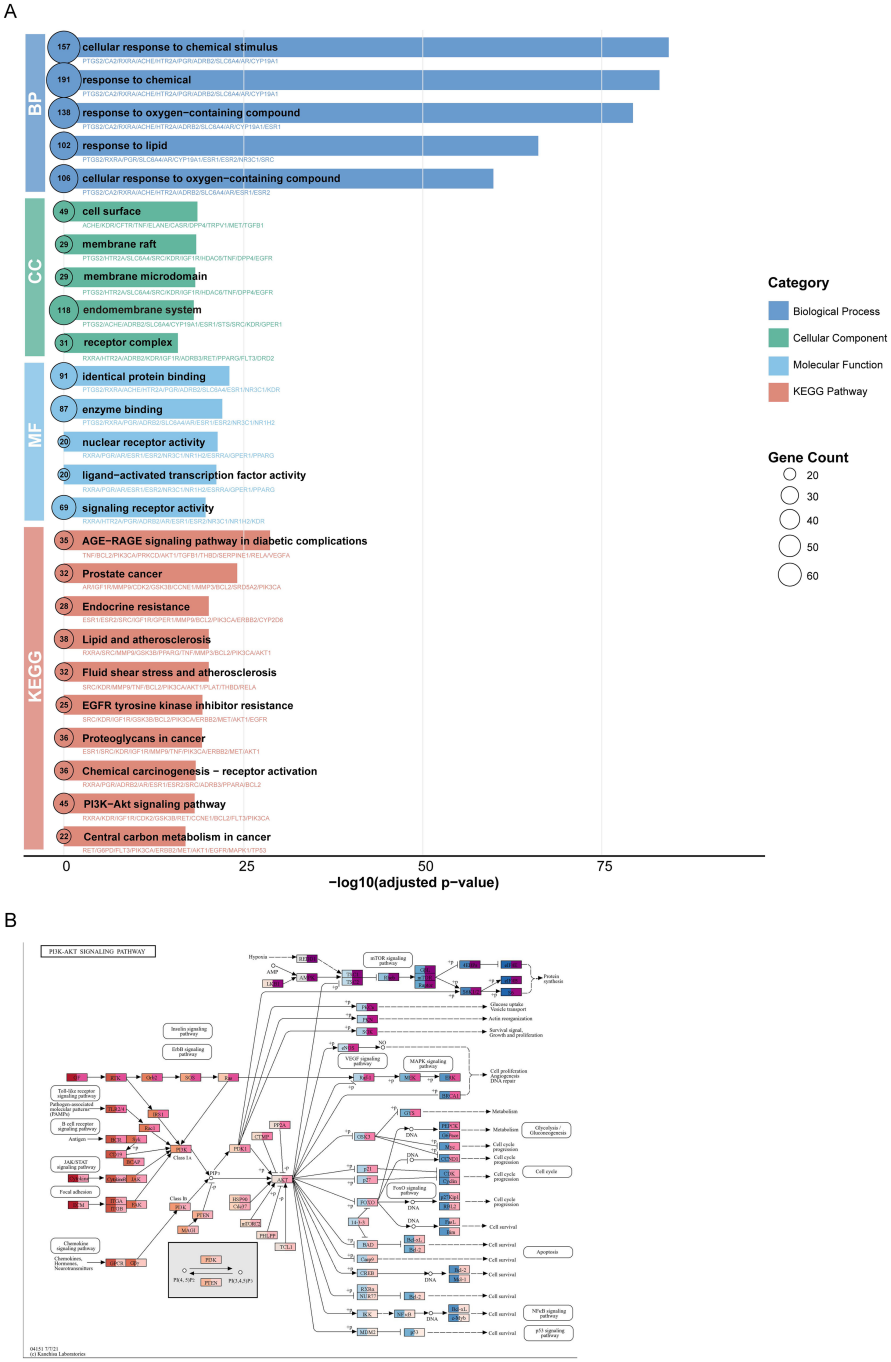
Pathway enrichment analysis of overlapping genes in PMOP. **(A)** GO enrichment analysis of overlapping genes, highlighting the top 5 terms in BP, CC, and MF categories, along with KEGG pathway enrichment analysis, displaying the top 10 enriched pathways. **(B)** KEGG pathway maps for the PI3K-AKT signaling pathway.

To explore the potential biological functions and therapeutic implications of the 235 shared targets associated with the SJP compound and PMOP, enrichment analyses based on GO annotations and KEGG pathways were conducted (Figure 7A). The GO analysis revealed significant enrichment across multiple functional categories. The most significantly enriched biological processes (BP) included terms such as cellular response to oxidative stress, lipid metabolic processes, and regulation of hormone levels. Regarding cellular components (CC), the target genes were predominantly localized to membrane rafts, vesicle lumens, and transcription regulator complexes. The KEGG pathway analysis further identified significant enrichment in pathways closely linked to PMOP. Notably, the well-established role of the PI3K-AKT signaling pathway in regulating bone cell survival, proliferation, and metabolic homeostasis underscores its central role as a mediator of the therapeutic effects of SJP against PMOP (Figure 7B). The results indicate that the overlapping genes exhibit diverse functions in metabolic control, intercellular communication, and inflammatory processes in PMOP.

### 3.8 Molecular docking and dynamics simulations of SJP compounds interacting with key metabolic enzymes

**Figure 8:**
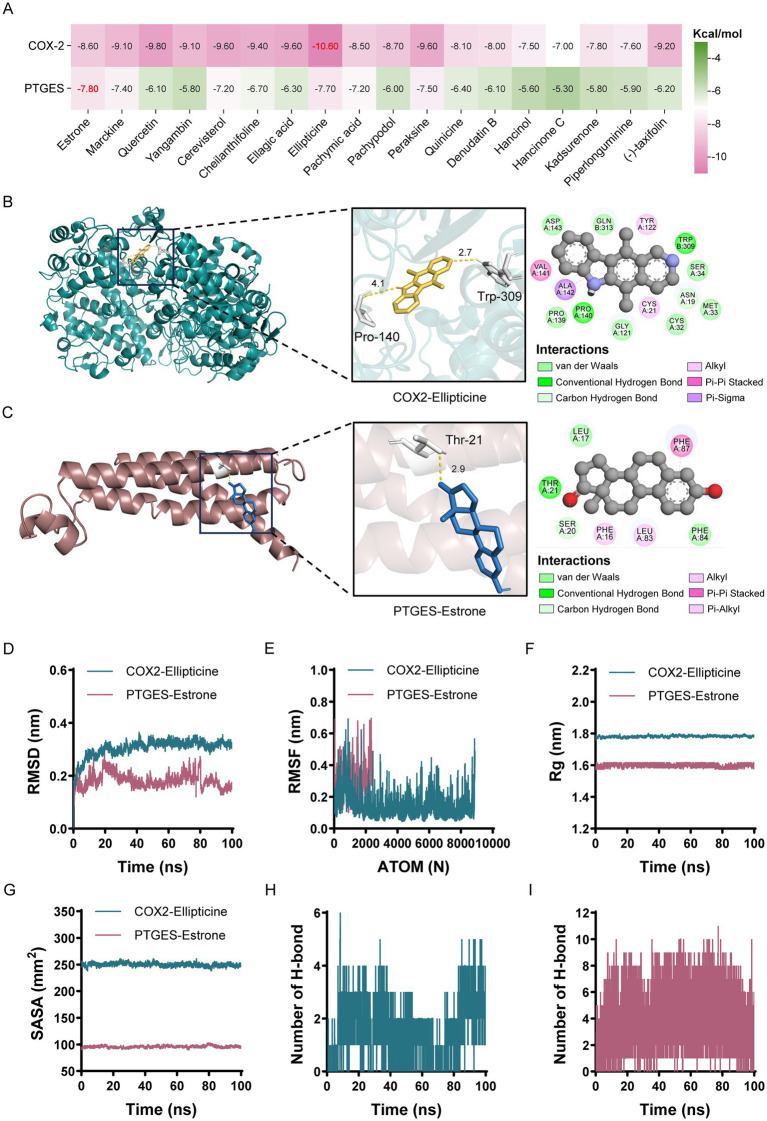
The active compounds of SJP interact with enzymes in the arachidonic acid metabolic pathway, specifically involved in the synthesis of prostaglandin E2. **(A)** Heatmap showing the molecular docking of COX2 and PTGES enzymes with SJP-activating compounds. **(B)** Docking model of COX2 with Ellipticine. **(C)** Docking model of PTGES with Estrone. **(D–I)** Molecular dynamics simulation results for the hub protein-ligand complexes. **(D)** RMSD changes over time during the simulation. **(E)** RMSF of the protein backbone. **(F)** Evolution of the Rg over time. **(G)** SASA variations in the protein-ligand complex during the simulation. **(H,I)** Number of hydrogen bonds formed within the COX2-Ellipticine and PTGES-Estrone complexes throughout the simulation.

To elucidate the mechanistic basis of SJP in PMOP through AA metabolism and PGE2 biosynthesis, we conducted molecular docking studies involving 18 active SJP constituents and two pivotal enzymes, COX2 and PTGES, which catalyze the conversion of AA to PGE2. The results revealed that Ellipticine exhibited the highest binding affinity for COX2 (−10.60 kcal/mol), while Estrone demonstrated the greatest affinity for PTGES (−7.80 kcal/mol) (Figures 8A–C). To assess the stability of the ligand-enzyme complexes, MD simulations were performed for the Ellipticine-COX2 and Estrone-PTGES complexes. RMSD profiles demonstrated rapid equilibration and sustained conformational stability, while low RMSF values at the binding sites indicated minimal fluctuation and strong ligand interactions (Figures 8D,E). These results were corroborated by Rg and SASA analyses, which revealed maintained structural compactness without significant unfolding, collectively demonstrating robust complex formation (Figures 8F,G and Table 3). Hydrogen bond occupancy analysis highlighted the critical role of persistent hydrogen bonds in supporting complex stability (Figures 8H,I). These high-affinity bindings suggest that Ellipticine and Estrone are promising candidates for modulating AA metabolism and PGE2 synthesis in PMOP.

**Table 3:** MMPBSA of enzyme-ligand (kcal/mol).

Energy type	COX2-ellipticine	PTGES-estrone
Δ*E_elec_*	−35.71	−9.37
Δ*E_vdW_*	−47.89	−7.24
Δ*G_PB_*	67.65	9.67
Δ*G*_SA_	−5.51	−0.98
-TΔS	12.09	16.49
Δ*G_bind_*	−35.71	−7.92

### 3.9 SJP mitigates OVX-induced intestinal barrier damage and activates EP4-PI3K/AKT to combat PMOP

**Figure 9:**
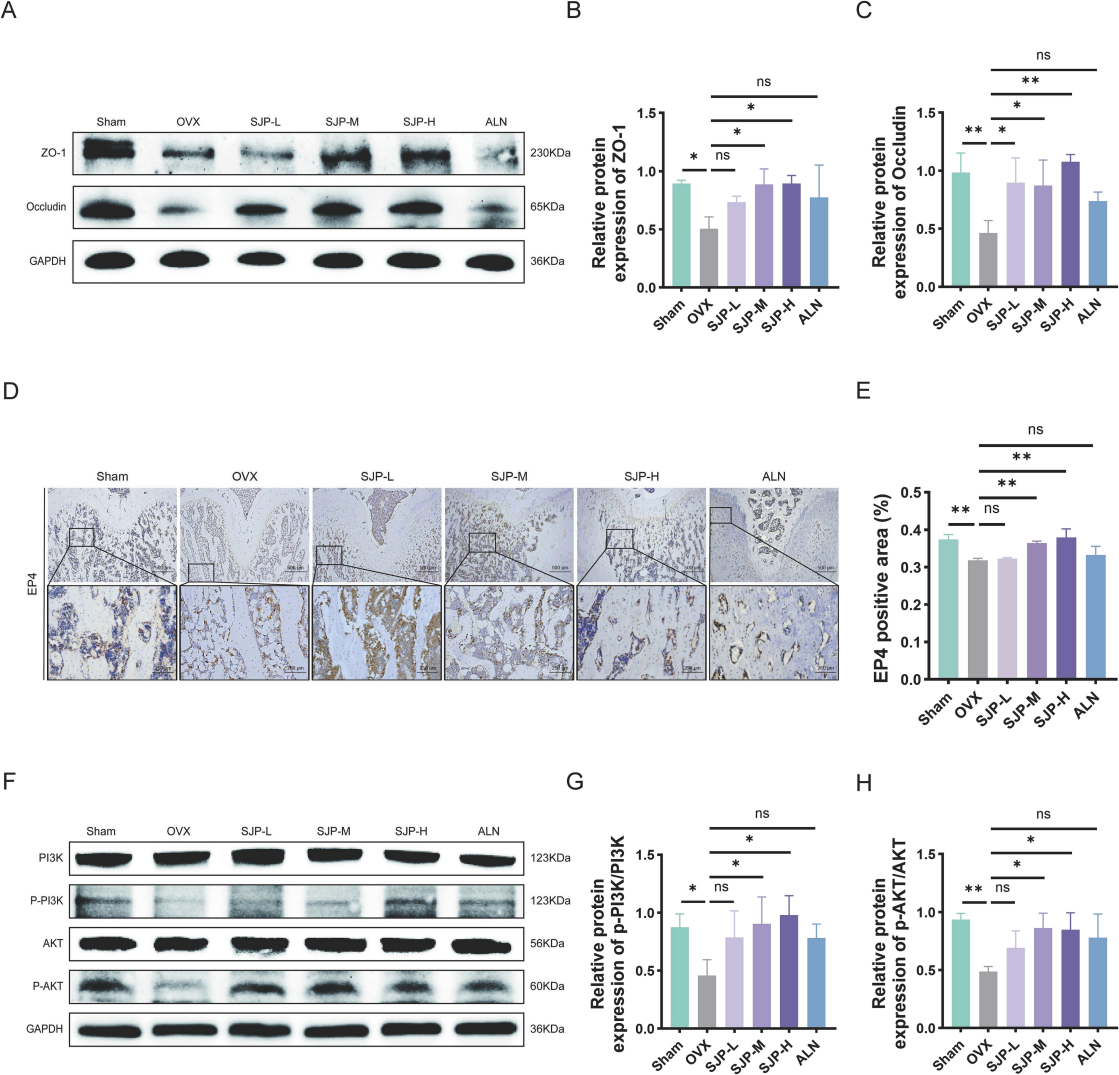
SJP enhances intestinal barrier function and activates the EP4-PI3K-AKT pathway in bone. **(A)** Representative protein expression bands for ZO-1 and Occludin. **(B)** Western blot analysis showing ZO-1 expression. **(C)** Western blot analysis showing Occludin expression. **(D)** Immunohistochemical staining of EP4 in femur. **(E)** Immunohistochemistry quantifying EP4 expression in the femur. **(F)** Representative protein expression bands for PI3K, AKT, p-PI3K, and p-AKT. **(G)** Western blot analysis of p-PI3K/PI3K expression. **(H)** Western blot analysis of p-AKT/AKT expression. Data are shown as mean ± SEM (*n* = 3). *p < 0.05, **p < 0.01.

To assess the impact of SJP on intestinal barrier integrity, we evaluated the expression levels of tight junction proteins, specifically ZO-1 and Occludin, in the ileal tissue. Compared to the Sham group, the OVX group showed a marked reduction in the expression of both ZO-1 and Occludin. In contrast, administration of SJP restored these levels in a dose-dependent manner, indicating the potential of SJP to maintain the integrity of the intestinal barrier (Figures 9A–C). Additionally, immunohistochemical analysis revealed a substantial decrease in EP4 expression within the bone tissue of OVX rats compared to the Sham group. Following SJP treatment, EP4 expression exhibited a dose-dependent increase, with the most substantial upregulation observed in the SJP-H group (Figures 9D,E). Considering the well-established role of the PGE2-EP4 axis in activating the PI3K-AKT signaling pathway, a crucial regulator of osteogenic differentiation, and the KEGG analysis results indicating significant enrichment and activation of this pathway subsequent to SJP treatment, we carried out additional investigations regarding the expression levels of PI3K, p-PI3K, AKT, and p-AKT through Western blot analysis. The findings showed that the phosphorylation levels of both PI3K and AKT were significantly lower in OVX rats compared to the Sham controls, suggesting that the PI3K-AKT signaling pathway is inhibited under conditions of estrogen deficiency. Following the SJP intervention, we observed a dose-dependent increase in the expression levels of p-PI3K and p-AKT, with the most pronounced effect noted in the SJP-H group (Figures 9F–H). In summary, our results demonstrate that SJP mitigates estrogen-deficiency-induced bone loss by concurrently enhancing intestinal barrier function and activating the osteogenic PGE2-EP4-PI3K/AKT cascade in bone.

### 3.10 Correlation between gut microbiota, bone markers, inflammatory factors, and EP4/PI3K-AKT pathway

To elucidate the mechanism of gut-bone interaction, a Spearman correlation analysis was conducted to ascertain the relationships between the composition of the gut microbiota and key components of relevant biological pathways. The study revealed a significant disparity in the microbial community structure between the SJP and OVX groups. The microbiota enriched in the OVX group, including *Lachnospiraceae_NK4A136_group*, *Escherichia_Shigella*, and *Haemophilus*, were associated with a cascade of detrimental physiological changes. We observed that alterations in gut microbiota within the OVX model were directly related to impaired barrier function. *Lachnospiraceae_NK4A136_group* exhibited a negative correlation with PGE2 (rho = −0.883, *p* = 0.003), while the abundance of *Escherichia_Shigella* was strongly inversely correlated with ZO-1 (rho = −0.867, *p* = 0.005). Given the critical role of the gut barrier in maintaining immune homeostasis, we systematically evaluated its association with systemic inflammation. The impaired gut barrier observed in the OVX group was linked to a pro-inflammatory state, as evidenced by strong positive correlations between OVX-enriched bacterial genera (*Escherichia_Shigella*, *Haemophilus*) and the pro-inflammatory cytokine TNF-α (rho = 0.867, *p* = 0.005).

**Figure 10:**
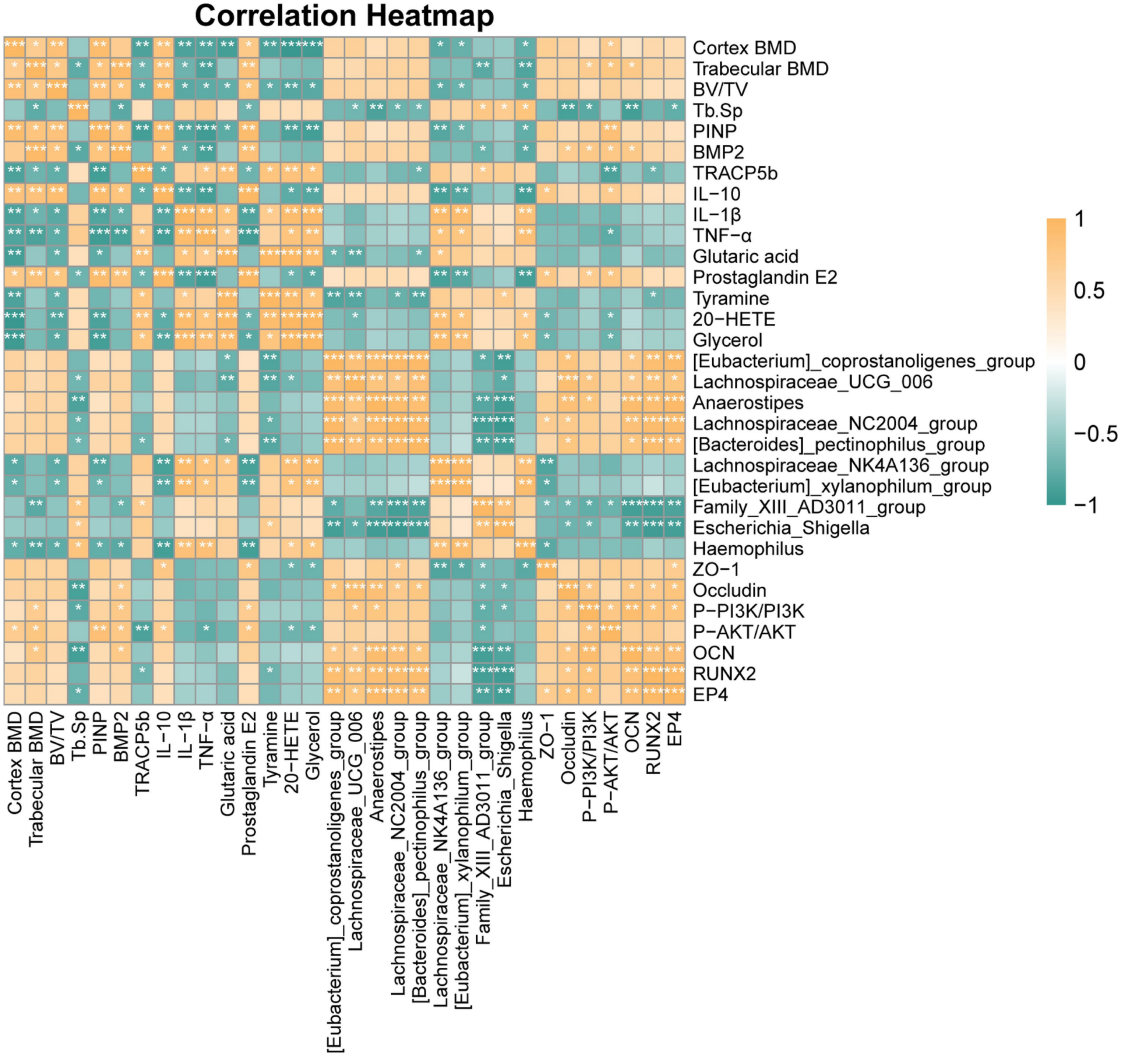
Heatmap depicting correlations between microbiome, differential metabolites, bone markers, inflammatory markers, and intestinal barrier markers across rat groups. Orange indicates positive correlations, while green represents negative correlations.

Systemic inflammation was further associated with impaired bone formation, as indicated by a significant negative correlation between serum TNF-α and the bone formation marker PINP (rho = −0.933, *p* < 0.001). In contrast, the SJP-modulated microbiota, particularly *Anaerostipes* and *[Eubacterium]_coprostanoligenes_group*, was associated with a protective signaling cascade. Specifically, the abundance of *Anaerostipes* strongly correlated with the upregulation of the PGE2 receptor EP4 (rho = 0.949, *p* < 0.001). Subsequently, we investigated the downstream signaling pathways and their osteogenic potential. The activation of the EP4 receptor positively correlated with the PI3K/AKT pathway (P-PI3K/PI3K: rho = 0.800, *p* = 0.014), which was critically linked to enhanced bone formation and demonstrated significant positive correlations with the key osteogenic transcription factor RUNX2 (rho = 0.800, *p* = 0.014) and the bone formation marker OCN (rho = 0.900, *p* = 0.002) (Figure 10 and Supplementary Figure S5). Collectively, these findings indicated that SJP treatment may prevent bone loss by promoting a favorable gut microbiota composition, enhancing PGE2 signaling, strengthening intestinal barrier function, reducing systemic inflammation, and activating the osteogenic EP4-PI3K/AKT signaling pathway, thereby fostering a microenvironment conducive to bone formation.

## 4 Discussion

The PMOP has emerged as a critical global public health challenge for the aging population (Brown, 2021; Subarajan et al., 2024). Primarily driven by postmenopausal estrogen deficiency, the disease is characterized by disrupted bone homeostasis (Wright et al., 2024; Yao et al., 2025). At the molecular level, this disruption manifests as a substantial imbalance between osteoblast formation and osteoclast development, ultimately resulting in decreased bone mineral density and an increased risk of fractures (Almeida et al., 2017; Zhang et al., 2025).

Sijing Pill, a traditional herbal formulation, is extensively utilized in clinical practice to warm and tonify the kidney and spleen, demonstrating therapeutic benefits in PMOP. Utilizing the well-established OVX rat model of PMOP, we found that 13 weeks of treatment with SJP conferred protection. Treatment with SJP ameliorated excessive body weight gain and significantly restored bone microarchitecture, as evidenced by increased BMD and BV/TV, along with a reduction in Tb. Sp to levels comparable to the Sham group. At the molecular level, SJP enhances the activity of crucial osteogenic transcription factors in bone, such as RUNX2 and OCN. Simultaneously, an increase in the levels of serum bone formation markers, PINP and BMP2, was observed, while the level of TRACP5b, a marker of bone resorption, significantly decreased in serum. Collectively, our findings establish the efficacy of SJP in mitigating PMOP, as evidenced by improvements in bone microstructure, molecular expression, and serum biomarkers. Notably, the SJP-H group outperformed ALN in elevating PINP and BMP2 levels, suggesting that SJP possesses superior osteogenic capacity and underscoring its promise as a complementary or alternative therapeutic strategy for PMOP.

Estrogen deficiency is a well-established contributor to gut microbiota dysbiosis in postmenopausal women (Xu et al., 2017; Guan et al., 2023; Kumari et al., 2024). Estrogen deficiency significantly alters gut microbiota structure, particularly the F/B ratio (Tu et al., 2020). Firmicutes dominance boosts energy harvest and alters short-chain fatty acid metabolism, weakening the intestinal barrier and triggering low-grade inflammation (Yuan et al., 2022; Wang et al., 2024). Meanwhile, reduced Bacteroidetes impairs bile acid metabolism and immune regulation (He et al., 2026). A higher F/B ratio correlates with increased energy harvest and worse intestinal inflammation (Wang et al., 2022; Yadav et al., 2023). Our findings align with clinical evidence, demonstrating that OVX in rats significantly altered the structure of the gut microbial community, as evidenced by an increased F/B ratio at the phylum level. At the genus level, OVX resulted in the enrichment of pathogenic taxa such as *Lachnospiraceae_NK4A136_group* and *Escherichia_Shigella*, both known to produce lipopolysaccharide (LPS) (Lei et al., 2024). Treatment with SJP effectively normalized the F/B ratio and increased the abundance of beneficial genera, including *[Eubacterium]_coprostanoligenes_group* and *Anaerostipes*, which are associated with SCFA production (Wei et al., 2021; Singh et al., 2023). Both alpha and beta diversity analyses demonstrated that SJP enhanced microbial abundance in the stomachs of mice in the OVX group and restored their species composition, resulting in a microbial profile highly similar to that of the Sham group. These results indicate that SJP contributes to the correction of gut dysbiosis induced by estrogen deficiency by restoring microbial balance, specifically by normalizing the F/B ratio and augmenting SCFA-producing bacteria, thereby underpinning its therapeutic efficacy against PMOP.

Gut microbiota dysbiosis is generally associated with alterations in the host metabolome (Singh et al., 2023). Non-targeted metabolomics analysis revealed 957 differentially expressed metabolites when comparing the OVX and Sham groups. Notably, SJP intervention reversed the expression of 633 of these metabolites, with 384 exhibiting opposing trends in the OVX and SJP-OVX groups. KEGG pathway enrichment analysis of these 384 metabolites identified arachidonic acid metabolism as a significantly regulated pathway. Specifically, SJP was shown to modulate the biosynthesis of PGE2, indicating its potential role in regulating lipid mediator production through microbial-metabolic interactions. This metabolic modulation, particularly within the arachidonic acid cascade, may represent a crucial mechanism underlying the anti-inflammatory and osteoprotective effects of SJP.

The stratification of metabolites into host-derived, microbiota-derived, and co-metabolized categories provides critical insights into the therapeutic mechanism of SJP. Specifically, AA metabolism, identified here as a prominent co-metabolized pathway, illustrates the intricate host-microbe interplay underpinning the disease phenotype. We observed a distinct metabolic shift in the OVX group, characterized by the accumulation of 20-HETE and the depletion of PGE2. Since 20-HETE acts as a potent vasoconstrictor and inducer of oxidative stress (Roman, 2002), its elevation aggravates bone loss (Wauquier et al., 2009; Manolagas, 2010); conversely, physiological levels of PGE2 are requisite for osteoblast differentiation and bone formation (Zhang et al., 2002; Blackwell et al., 2010). This maladaptive metabolic profile in OVX mice showed a significant correlation with the enrichment of potentially pathogenic genera, such as *Escherichia-Shigella* and *Haemophilus*. These taxa may exacerbate systemic inflammation (Shin et al., 2015), thereby favoring the CYP450-mediated conversion of AA to 20-HETE (Theken et al., 2011). Treatment with SJP effectively reversed this dysregulation, lowering 20-HETE levels while restoring PGE2 to physiological concentrations. Notably, this metabolic restoration was accompanied by an increase in beneficial SCFA-producing bacteria, including *Anaerostipes*, the *Eubacterium_coprostanoligenes_group*, and *Lachnospiraceae_UCG-006* (Louis and Flint, 2017; Vacca et al., 2020). Given that SCFAs attenuate inflammation and reinforce gut barrier integrity (Parada Venegas et al., 2019), SJP likely functions by remodeling the gut microbiota to redirect AA metabolic flux from the pathological 20-HETE axis toward the homeostatic PGE2 pathway. Consequently, the concept of co-metabolism highlights that the therapeutic efficacy of SJP relies on rectifying the gut-driven dysregulation of host metabolism.

Gut dysbiosis and the associated metabolic disturbances may impair intestinal barrier function (Hu et al., 2022; Fujisaka et al., 2023). The integrity of the intestinal barrier, maintained by tight-junction proteins such as occludin and ZO-1, is crucial for preventing the migration of harmful substances from the gut lumen into the bloodstream (Fujisaka et al., 2023). The results of the study indicated that rats subjected to OVX exhibited significantly reduced levels of ZO-1 and occludin expression, suggesting compromised barrier integrity. Notably, the administration of SJP restored the expression of these proteins, indicating a protective effect on the intestinal barrier. These findings suggest that SJP enhances gut barrier function, at least in part, through microbiota-derived short-chain fatty acids, thereby mitigating the risk of a “leaky gut” phenotype.

Damage to the intestinal barrier allows bacterial toxins, such as lipopolysaccharide, to enter the bloodstream, potentially initiating a systemic inflammatory response (Hammed et al., 2025; Mao et al., 2025). Consistent with this mechanism, our ELISA data revealed a systemic pro-inflammatory state in OVX rats, characterized by elevated levels of TNF-α and IL-1β, alongside diminished IL-10. In contrast to the OVX group, the administration of SJP normalized the disrupted cytokine profile, suppressing TNF-α and IL-1β while increasing IL-10. This coordinated immunomodulation, likely stemming from the restoration of gut barrier integrity, interrupts the vicious cycle of inflammation-mediated bone loss by rebalancing bone remodeling signals, thereby uncoupling gut dysbiosis from skeletal deterioration.

To systematically elucidate the multi-target mechanism of SJP, an integrative approach combining network pharmacology and computational simulations was employed. Network pharmacology, with its capacity to address complex biological interactions, is particularly well-suited for uncovering the intricate mechanisms of herbal formulations (Leem et al., 2022). KEGG pathway analysis of the network pharmacology targets revealed significant enrichment in the PI3K-AKT signaling pathway, a well-established regulator of osteoblast survival and differentiation. Western blot analysis demonstrated that OVX suppressed the phosphorylation of PI3K and AKT, whereas SJP treatment dose-dependently enhanced the levels of p-PI3K and p-AKT. Additionally, immunohistochemical results indicated that SJP upregulated the expression of the EP4 receptor, a key PGE2 receptor known to activate the PI3K-AKT signaling cascade upon ligand binding (George et al., 2007; Yokoyama et al., 2013; Xu et al., 2018). These data support a functional signaling cascade wherein SJP-mediated modulation of PGE2 activates the EP4 receptor, initiating the downstream PI3K-AKT pathway. The SJP-PGE2-EP4-PI3K-AKT signaling axis delineates a coherent, multi-target mechanism for the action of SJP in PMOP, enhancing osteoblast activity and mitigating bone loss, thereby establishing a direct functional link from gut microbiota modulation to the activation of anabolic signaling in bone.

This study systematically elucidates the mechanisms by which SJP exerts its therapeutic effects on the gut-bone axis. Nevertheless, several limitations must be acknowledged. Most notably, the absence of fecal microbiota transplantation experiments precludes a definitive establishment of causality. Furthermore, while our data strongly implicate the PGE2-EP4-PI3K-AKT signaling pathway, its specific role demands functional validation, ideally through pharmacological inhibition or genetic knockout models.

## 5 Conclusion

This study established that the traditional herbal formulation SJP mitigated PMOP in an OVX rat model through a multi-scale mechanism mediated by the gut-bone axis. We revealed that SJP not only directly attenuated bone loss by restoring trabecular microstructure and enhancing bone mineral density but also systemically ameliorated OVX-induced metabolic disturbances and gut microbiota dysbiosis. In summary, these findings position SJP as a multi-system therapeutic candidate for PMOP and underscore the SJP-PGE2-EP4-PI3K-AKT signaling axis as a compelling target for further research into metabolic bone disorders.

## Data Availability

The datasets presented in this study can be found in the Sequence Read Archive at the NCBI under BioProject accession number PRJNA1433527.

## Footnotes

1
https://www.tcmsp-e.com/

2
http://herb.ac.cn/

3
http://bionet.ncpsb.org.cn/batman-tcm/

4
http://www.swisstargetprediction.ch/

5
https://www.genecards.org/

6
https://www.omim.org/

7
https://www.pharmgkb.org/

8
https://db.idrblab.net/ttd/

9
https://cn.string-db.org/

10
http://metorigin.met-bioinformatics.cn/

11
https://www.rcsb.org/
